# Primitive icosahedral quasicrystals in ZnMgLi(Dy, Ho, Er, Tm) systems

**DOI:** 10.1107/S205327332501099X

**Published:** 2026-01-22

**Authors:** Ireneusz Buganski, Stanislav Vrtnik, Radoslaw Strzalka, Andreja Jelen, Sandra Drev, Janusz Wolny, Nobuhisa Fujita

**Affiliations:** a Faculty of Physics and Applied Computer Science AGH University of Krakow Al. Mickiewicza 30 Krakow 30-059 Poland; b Jožef Stefan Institute Jamova 39 Ljubljana SI-1000 Slovenia; c Faculty of Mathematics and Physics University of Ljubljana Jadranska 19 Ljubljana SI-1000 Slovenia; d Institute of Multidisciplinary Research for Advanced Materials Tohoku University Sendai 980-8577 Japan; Université de Lorraine, France

**Keywords:** quasicrystals, crystal growth, Bergman phase, *ab initio* structure solution

## Abstract

New quasicrystals with rare-earth elements and Li are synthesized with the self-flux method. The starting composition involves 62.8 at.% Zn, 28.6 at.% Mg, 3.6 at. % rare-earth and 5% Li. Alternatively, 66 at.% Zn, 24.8 at.% Mg, 3.9 at.% rare-earth and 5.3% Li can be used. Both syntheses result in quasicrystals exhibiting differences in the quasilattice constant which is additionally correlated with the size of the rare-earth atom. Monocrystal X-ray diffraction reveals that Li substitutes the rare-earth element found in the center of the Tsai cluster. The substitution of Li for the rare-earth element not only changes the electron-to-atom ratio but also changes the distribution of magnetic-moment-bearing elements which can affect magnetic properties.

## Abstract 2

The study introduces new primitive icosahedral quasicrystals in Zn–Mg–Li–(Dy, Ho, Er, Tm) systems. Quasicrystals are studied with an X-ray diffraction technique resolving the local atomic structure.

## 1. Introduction

Long-range magnetic order, both ferromagnetic and antiferromagnetic, was discovered in Tsai-type icosahedral quasicrystals. The ferromagnetic order was confirmed through magnetic susceptibility measurements and neutron diffraction in Au_65_Ga_20_(Tb, Gd)_15_ phases (Tamura *et al.*, 2021 ▸). The antiferromagnetic state was identified in Au_56_In_28.5_Eu_15.5_ (Tamura *et al.*, 2025 ▸). Both discoveries are the result of two decades of research dedicated to determining the magnetic properties of icosahedral quasicrystals in the Tsai-type family. Over time, it was established that the type of magnetic ordering is dictated by the electron-to-atom ratio (*e*/*a*) (Suzuki *et al.*, 2021 ▸). All investigated periodic approximant crystals could be represented on a common curve, where the Curie–Weiss temperature normalized by the de Gennes factor was plotted against *e*/*a*. It was determined that non-spin-glass order could be expected for *e*/*a* values below 1.8. Unfortunately, magnetically ordered quasicrystals discovered so far cannot be grown by slow solidification and must instead be obtained either through the melt-spinning technique or arc melting.

Before the discovery of the Tsai-type family, Bergman-type icosahedral quasicrystals in Zn–Mg–RE (RE = rare-earth) systems were extensively studied for their magnetic properties. The first member of this family was obtained by Luo *et al.* (1993 ▸) in the Mg_76.99_Zn_7.61_Y_15.4_ phase. This discovery inspired further research in which various RE elements were substituted for Y, greatly expanding the number of known icosahedral phases (Niikura *et al.*, 1994*a* ▸; Niikura *et al.*, 1994*b* ▸). Magnetic property measurements became possible thanks to the development of reliable experimental procedures for producing large single crystals via the self-flux method. The method was refined for face-centered icosahedral (FI) phases by Fisher *et al.* (1998 ▸). Later, the growth conditions for primitive icosahedral (PI) phases were determined in a series of studies by Langsdorf & Assmus (1998 ▸), Sterzel *et al.* (2000 ▸), Uhrig *et al.* (2003 ▸) and Uhrig *et al.* (2005 ▸). Despite these efforts, only spin-glass transitions could be confirmed in these systems (Fisher *et al.*, 1999 ▸; Sato, 2005 ▸; Sato & Tsai, 2007 ▸; Goldman, 2014 ▸).

The motivation for the present study was to obtain new quasicrystals in the Zn–Mg–RE family, with the *e*/*a* ratio controlled by the concentration of a fourth element. By tuning the effective Fermi vector in RKKY (Ruderman–Kittel–Kasuya–Yoshida) interactions through doping with an element of lower valency, it might be possible to induce magnetic ordering, as suggested by the study of Suzuki *et al.* (2021 ▸). We chose Li(I) with the aim of substituting Mg(II) in the formula. The substitution of Mg with Li had already been utilized by Lee & Miller (2001 ▸) in their study of preferential occupancy of crystallographic sites in the 1/1 periodic approximant crystal of the Al–Zn–Mg system. According to their findings, element interchange occurs primarily in the most electropositive sites, qualitatively determined via Mulliken partial charges. Preferential site occupancy depending on electronegativity was also confirmed for the Tsai-type ZnMgSc 1/1 periodic approximant crystal (Buganski *et al.*, 2024*b* ▸) and for another Zn-based Bergman-type 1/1 ZnMgHf periodic approximant crystal (Buganski *et al.*, 2025 ▸). In light of all this research, the substitution was predicted to be feasible.

## 2. Crystal growth

Single crystals were prepared using the self-flux method. High-purity elements were weighed and placed in an alumina crucible. The elements were used in the following forms: Zn (4N), pieces up to 1 g; Mg (4N), pieces up to 0.2 g; RE (3N), pieces up to 0.3 g; Ho, we used both pieces up to 0.3 g and metal turnings (<0.1 g); and Li (4N), pieces of ∼0.015 g cut with scissors from a bar of 1 cm diameter. The total weight of elements did not exceed 5 g.

Two main starting compositions were employed, both yielding high-quality single crystals. The first was adapted from Uhrig *et al.* (2005 ▸): Zn_62.8_Mg_28.6_RE_3.6_Li_5_, where 5 at.% Mg was replaced with Li. The second composition was Zn_66_Mg_24.8_RE_3.9_Li_5.3_. Both produce PI-type quasicrystals. Slight variations of the Li/Mg ratio were also tested.

After weighing, the crucible was sealed in a quartz tube under argon at 600 mbar. Heat treatment, following the procedure described by Uhrig *et al.* (2005 ▸), was carried out in a muffle furnace (model FO200, Yamato Scientific Co., Ltd). The sample was heated from room temperature to 750°C within 1 h, kept at this temperature for 5 h to melt, and then cooled to 590°C over 80 h. The final step was annealing at 590°C for 3.5–140 h. After annealing, the quartz ampoules were removed from the furnace and centrifuged at 1500 rev min^−1^ for 15 min, separating the residual melt from the crystal grains. The samples used in this study are listed in Table 1 ▸.

**Figure 1:**
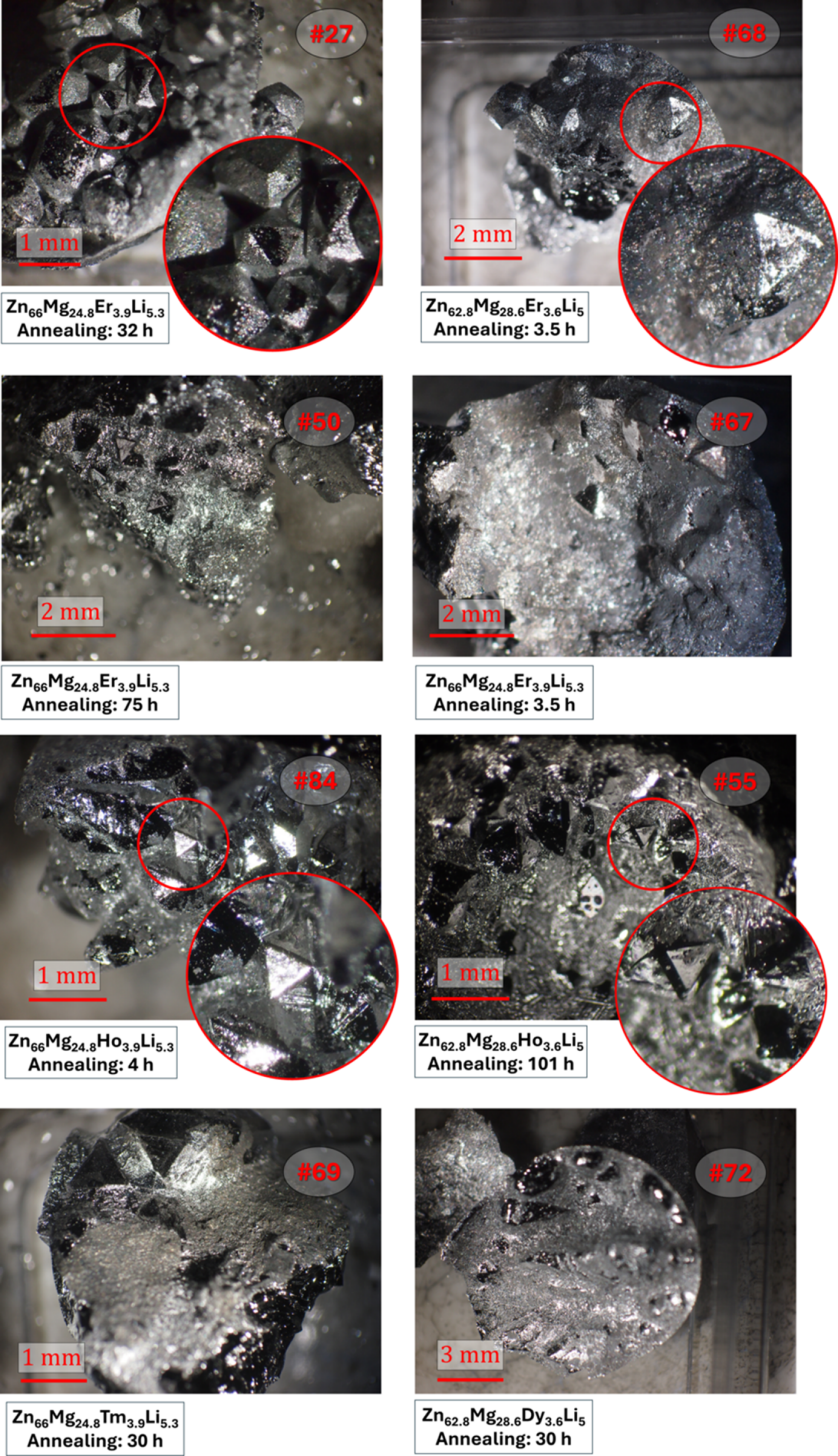
Optical microscopy photos of selected samples grown with the self-flux method. Single grains in the shape of an icosahedron indicate the presence of quasicrystals containing Li. Sample numbers according to in-laboratory metadata.

Crystal morphology was examined under an optical microscope. Unexpectedly, many single grains exhibited nearly perfect icosahedral shapes. From prior experience, FI-type ZnMgRE quasicrystals grown by the self-flux method usually form dodecahedral grains (Fisher *et al.*, 1998 ▸; Niikura *et al.*, 1994*a* ▸), while PI ZnMgRE quasicrystals show rhombic triacontahedral grains as shown in Fig. S1 (in the supporting information). The presence of icosahedral grains thus provided the first evidence for new quasicrystalline phases. Fig. 1 ▸ shows camera images of the exemplary synthesized ZnMgRELi quasicrystals and Table 1 ▸ presents a list of considered samples exhibiting large single grains.

**Figure 2:**
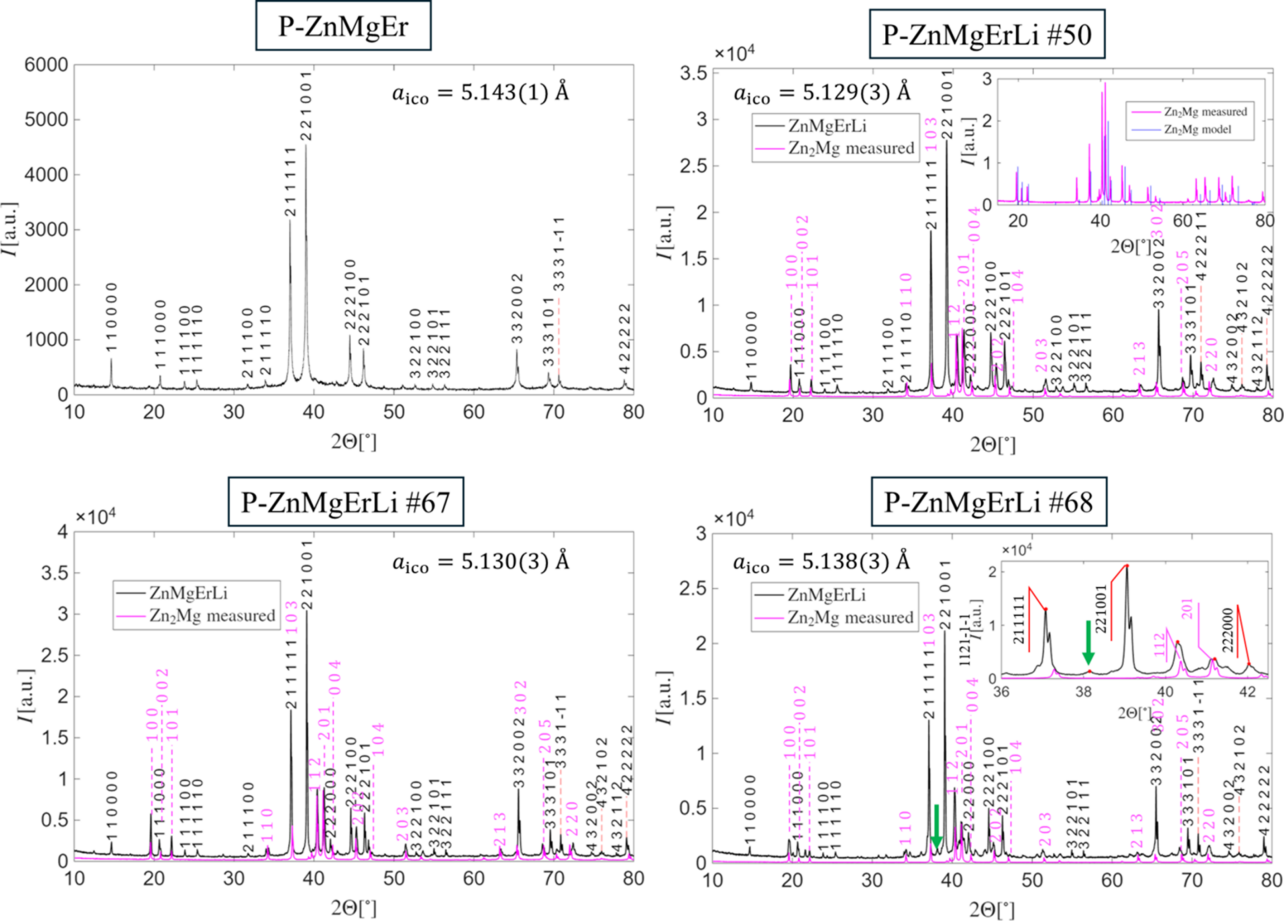
The PXRD pattern of selected ZnMgErLi samples including quasicrystals. Zn_2_Mg phase is present in all samples. The decrease of the lattice constant compared with ZnMgEr indicates the presence of Li in the quasicrystal. Indices of the quasicrystalline peaks follow the Elser setting of six indices whereas peaks related to Zn_2_Mg are given as three integers.

The clearest icosahedral grain was observed in sample #27 with Er, with a diameter of ∼1 mm. Most other samples, except #72 with Dy, exhibited grains with triangular faces, as expected for an icosahedron. Sample #72 contained numerous grains >1 mm, but none showed polyhedral shapes with icosahedral symmetry. However, powder X-ray diffraction (PXRD) confirmed the presence of quasicrystals (discussed in Section 3). Grain size appeared unaffected by annealing time, as presented in the photos in Fig. 1 ▸ in the second row. Two samples, #50 and #67, grown from the same initial composition with the same cooling rate but differing in annealing time (one was annealed for 3.5 h and the second for 75 h), have the same shape and size of grains. Additionally, the PXRD patterns shown in Fig. 2 ▸ for both samples do not show differences in peak sizes or broadening due to lower crystallinity.

We did not test how cooling rate affects the size of grains. However, we additionally tested the impact of variation of the initial content of Li on grain size and morphology. Tests were carried out for samples with Er and Ho. Sample #56, grown with 3 at.% Li, showed rhombic faces in the central grain, while larger grains at the sample edge lacked symmetric polyhedral morphology. Sample #35, with 10 at.% Li, displayed triangular faces, and its lattice constant was statistically indistinguishable from those of samples with 5 at.% Li. The small content of Li at the level of 3 at.% did not change the morphology of grains with respect to the PI phase in the ZnMgRE system. From this preliminary study, it can be stated that the larger content of Li, starting from 3 to 5 at.%, causes the slow growth along threefold symmetry axes, as evident by the presence of triangles in the resulting grains. The PXRD pattern and sample images are provided in the supporting information (Fig. S2).

The change in the crystal morphology with respect to Li content is an interesting feature. Morphological shapes of quasicrystalline grains have been the subject of earlier theoretical work (Ho *et al.*, 1987 ▸; Chattopadhyay *et al.*, 1997 ▸). It is generally accepted that the equilibrium crystal shape, determined by surface energy, may differ from the growth morphology due to kinetic effects. Predominant quasicrystal shapes are typically triacontahedra, dodecahedra or edge-roughened variants of these. Recent Wulff shape calculations for crystalline CdYb and ScZn also do not predict icosahedra as possible morphologies for quasicrystalline grains (Baek *et al.*, 2025 ▸). This issue warrants further study, as it may provide additional insight into the mechanisms of quasicrystal stabilization.

## 3. Powder X-ray diffraction and differential thermal analysis

PXRD data were collected using a Rigaku Ultima IV diffractometer equipped with a Cu lamp and D/teX Ultra detector. The *K*α line was used for data collection. For each measurement, a small amount of sample was crushed in a mortar, and the resulting powder was sieved through a 25 µm mesh.

Indexing was performed using the Elser setting (Elser, 1986 ▸). A peak was accepted when the difference between experimental and theoretical positions did not exceed 0.25°. If multiple solutions satisfied this condition, the one with the smaller perpendicular-space vector length was chosen. Peak 211111 was used as the reference to calculate 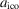, the icosahedral lattice constant. After indexing, 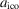 was refined by linear regression of theoretical versus experimental peak positions. Since multiple phases occur in the PXRD pattern, peaks for which the perpendicular-space wavevector was higher than 0.14 Å^−1^ remained unindexed. The threshold was chosen based on a PI ZnMgEr sample for which a low level of impurities is visible.

Fig. 2 ▸ compares PXRD patterns of selected ZnMgErLi samples with those of PI-type ZnMgEr quasicrystals synthesized according to the method of Uhrig *et al.* (2005 ▸). In all ZnMgErLi samples, additional phases were detected, primarily the Zn_2_Mg Laves phase. This assignment was confirmed when the Zn_2_Mg diffraction pattern was obtained in a failed synthesis, where its presence was verified by single-crystal diffraction and crystal morphology (hexagonal needles). For reference, the theoretical PXRD pattern of Zn_2_Mg was calculated with *VESTA* version 4.6.0 (Momma & Izumi, 2011 ▸) (inset in the top-right chart in Fig. 2 ▸). The intensity of Zn_2_Mg reflections occurring in the PXRD pattern of a quasicrystal was scaled based on the peak indexed as 201, which could not be indexed within the icosahedral setting. Several further unindexed reflections were present, indicative of other unidentified impurities.

In spite of restrictions in indexing, there are differences in the indexing of one of the peaks between samples #50, 67 and 68: peak 422211 in sample #50 is indexed as 333111 in other samples. The proper indices cannot be resolved purely by PXRD as for both those indices the perpendicular-space wavevector is much lower than the threshold (∼0.068 Å^−1^). It is possible that the peak is merged from both 333111 and 422211.

Li doping resulted in a measurable contraction of the lattice constant, despite the low Li content. For example 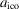 = 5.143 (1) Å for PI ZnMgEr (#22), and decreased to 5.130 (3) Å (#67) by doping with 5 at.% Li. Structural changes were also reflected in peak intensities: the height of the 332002 peak increased markedly in ZnMgErLi samples, whereas the 110000 peak height decreased. In some cases, reflections that could be indexed with the icosahedral setting overlap with peaks from Zn_2_Mg. For instance, peak 111000 overlaps with 002 and 211111 overlaps with 103. Other peaks like 201 originated entirely from Zn_2_Mg. Conversely, not all peaks could be explained just by a mix of quasicrystalline and Zn_2_Mg provenance. For example, peak 112 in the sample #67 perfectly matches the theoretical intensity of the Zn_2_Mg phase. However, in sample #68 the peak is much higher, elevated above the peak 201. That means the additional phase is present in the sample #68 or Laves phases in both samples differ in the composition. It requires further confirmation.

Samples #67 and #68, obtained from different starting compositions, exhibit qualitative differences associated not only with the impurity content. Sample #68 contains substantially less Zn_2_Mg than #67 but shows the presence of the additional phase marked by the elevated height of the peak 112. The icosahedral lattice constant differs significantly, consistent with later observations that Zn-richer compositions yield smaller lattice sizes. This might be related to lower Mg content in the quasicrystal as Mg is a larger atom than Zn. Annealing time did not appear to influence impurity content: for example, samples #50 (75 h anneal) and #67 (3.5 h) exhibit nearly identical patterns. Due to the presence of impurities, phason strain analysis was not attempted as both the width and locations of peaks are skewed by additional phases.

An intriguing feature was an additional peak observed between the 211111 (in the fivefold direction) and 221001 (in the twofold direction) reflections marked with a green arrow in Fig. 2 ▸. This peak appears only in Zn-depleted samples, such as #68, and could not be indexed with the icosahedral basis, indicating an impurity phase. It cannot be attributed to Zn_2_Mg either. Its occurrence is correlated with a strong enhancement of the peak identified as the 112 peak, beyond what can be attributed to Zn_2_Mg. This feature may signify the presence of a periodic approximant crystal, as those phases often produce additional reflections in this exact position (*e.g.* Takeuchi, 2009 ▸). In addition, for sample #35, we observe a few strong peaks that could not be indexed (Fig. S2). They are absent in remaining samples. This is possibly related to the higher concentration of Li in the starting composition reaching 10 at.%. The additional impurity phase could therefore be related to periodic phases with Li. In the ZnMgRE system, several approximant phases are known, including the rhombohedral E-phase in Zn_16.2_Mg_0.8_Gd_2.0_ (Uhrig *et al.*, 2005 ▸) and the P-phase in Zn_66_Mg_33_Ho_3_ (Sterzel *et al.*, 2000 ▸). At this time the detailed phase identification has not been performed as it is complicated by Li, which cannot be quantified by standard wavelength-dispersive X-ray analysis. The use of alternative techniques like atom probe tomography (Gault *et al.*, 2021 ▸) or energy-dispersive spectroscopy with a special detector must be considered for that purpose.

**Figure 3:**
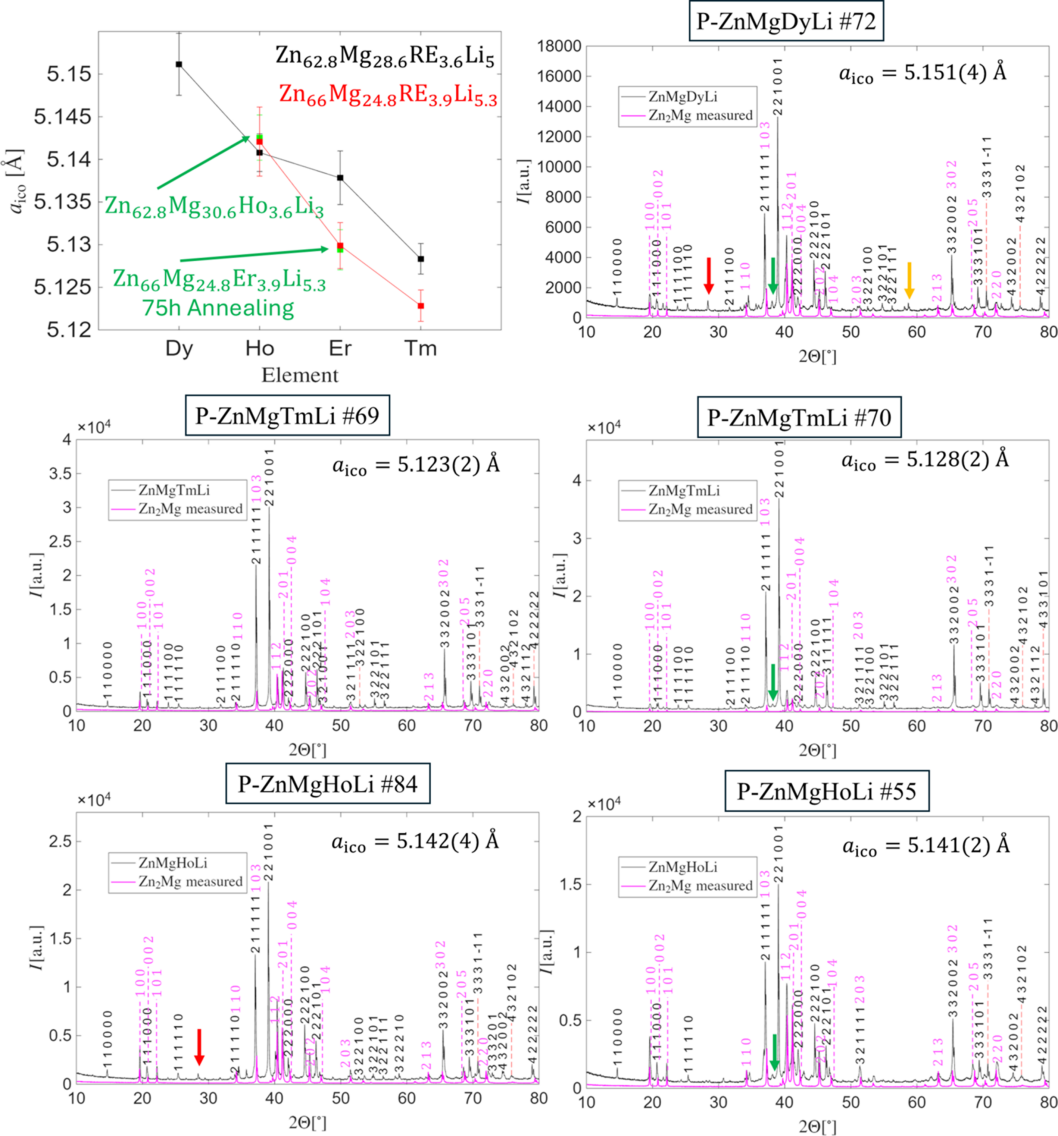
The PXRD pattern of ZnMg(Dy, Ho, Tm)Li quasicrystals containing Zn_2_Mg and other impurities. The icosahedral lattice size depends on both the type of RE element and the starting composition. Indices for the quasicrystal and Zn_2_Mg are given. Peaks that could not be indexed are marked by different colored arrows.

Fig. 3 ▸ shows PXRD patterns of samples with Dy, Ho and Tm. For Ho and Tm, the PXRD pattern for the Zn-rich phase is shown on the left-hand side, while the Zn-depleted phase is on the right-hand side. In each case, the presence of an icosahedral phase is confirmed by the appearance of the characteristic duo of 211111 and 221001 reflections. The 332002 peak remains significantly stronger than in ZnMgEr, which can be considered a marker of Li-containing quasicrystals. The amount of Zn_2_Mg impurity is governed by the starting composition, with Zn-richer samples exhibiting higher impurity levels. Sample #70 with Tm is nearly free of Zn_2_Mg, making the effect of Li on the PXRD pattern of the quasicrystal especially clear.

For Ho-containing phases, both samples #55 and #56 were prepared with 62.8% Zn, but #56 contained only 3% Li. As expected for Li-containing phases, the 332002 peak is enhanced, while the Zn_2_Mg impurity content is relatively low. The lattice constants of #55 and #56 are statistically identical, indicating no significant structural difference between both phases. The initial content of Li seems to not affect the final composition of the quasicrystal, indicating Li substitutes selected crystallographic sites – when saturated, no more Li is accepted in the structure.

Sterzel *et al.* (2000 ▸) reported that the liquidus temperature in the ZnMgY system depends on Zn content, and a similar dependence on Li may exist for ZnMgRELi. This suggests that to obtain single-phase samples, the target growth temperature must be adjusted according to composition. Supporting this view, the Dy-containing sample with a Zn-rich initial composition did not yield a quasicrystal under the same conditions. Dy is the largest RE element used in this study, and Niikura *et al.* (1994*b* ▸) showed that increasing RE atomic radius strongly affects quasicrystal stability; for even larger RE atoms, only amorphous phases are obtained.

PXRD data indicate a correlation between the icosahedral lattice constant and the atomic radius of the RE element: larger RE atoms lead to larger lattice constants, as plotted in the top-left of Fig. 3 ▸. In addition, Zn-depleted compositions consistently produce larger lattice parameters until Ho, suggesting an underlying structural difference. The Zn-rich starting composition used for Ho in sample #84 resulted in the same type of PXRD pattern and icosahedral lattice constant as for the Zn-depleted composition (Fig. 2 ▸). It is interesting that the additional peak found between 211111 and 221001 peaks in remaining Zn-depleted samples is not found in the PXRD pattern of sample #84 even though the height of the peak 112 is elevated. That fact makes them independent phenomena. Detailed clarification of these issues will require further study and full structure determination.

As well as the additional peak marked with a green arrow (in Fig. 3 ▸) for all Zn-depleted phases, some additional peaks can be found, as indicated by different colored arrows in Fig. 3 ▸, especially for the sample with Dy. Additional peaks are sharp but do not correspond to quasicrystals or Zn_2_Mg phase. The peak marked by a red arrow can also be found in the Ho-containing sample, but the peaks marked with a yellow arrow are restricted to the Dy-containing sample.

To clarify the origin of the additional peak between the two strongest reflections in Zn-depleted samples, we performed differential thermal analysis (DTA). Portions of ZnMgTmLi samples #69 and #70 were placed in alumina crucibles and measured under an Ar atmosphere up to 700°C, with an empty crucible as the reference. Two experiments were carried out for each sample. In the first, samples were heated and cooled at a constant rate of 10 K min^−1^. In the second, performed on a different portion of each sample, heating was conducted at 10 K min^−1^ up to 500°C and then at 5 K min^−1^ from 500 to 700°C, to better capture transitions in slowly forming phases.

**Figure 4:**
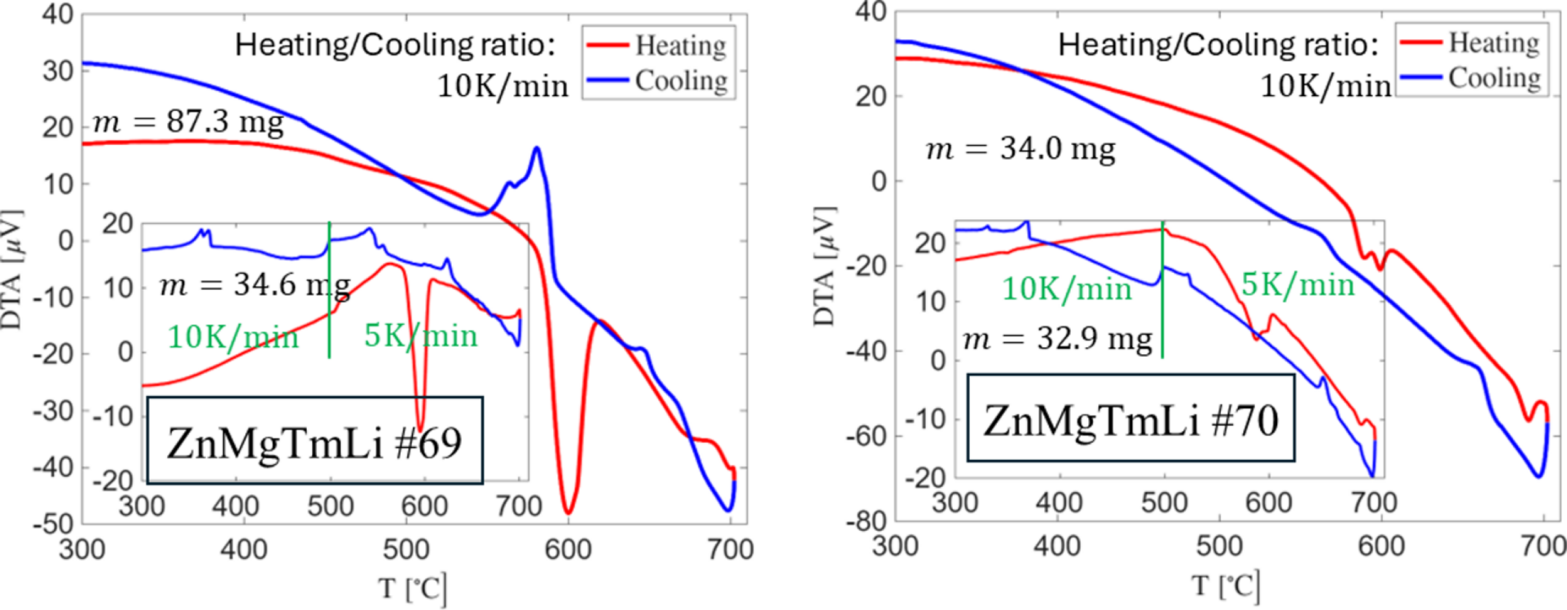
DTA signal for two samples with RE = Tm. For both samples the same two peaks occur around 578°C.

Fig. 4 ▸ presents the DTA curves for both samples. No conclusive evidence of an additional phase in sample #70 can be derived from the data. However, the small height of the peak around 580°C suggests a lower impurity fraction, related to Zn_2_Mg, consistent with the PXRD results. The peak is bimodal indicating the presence of an additional phase. The phase transition temperature corresponds well to the P- and K-phases reported by Sterzel *et al.* (2000 ▸). Quasicrystal formation occurs near 650°C, with a weak associated signal, particularly during cooling. The only notable difference between the two samples is a weak high-temperature signal close to 700°C in sample #70, absent in #69. This may indicate the formation of an additional phase, potentially responsible for the enhanced intensity of the 112 reflection and the extra unindexed peak between the strongest PXRD reflections.

Upon cooling, several peaks appear below 400°C, attributable to ZnMg phases formed during sample decomposition due to Zn loss. As much as 10 mg of material can be lost through Zn evaporation during heating close to 700°C. That is the main reason the experiment was not performed at higher temperatures.

## 4. Monocrystal X-ray diffraction

Single-crystal X-ray diffraction was performed on ZnMgErLi sample #27 and compared with the PI ZnMgEr quasicrystal. Data reduction was carried out using *CrysAlis PRO* v42.49 (Agilent, 2011 ▸) within the icosahedral 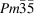 space group. For ZnMgEr, 4740 diffraction peaks were identified in the irreducible part of reciprocal space, of which 3359 satisfied the condition 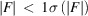. For ZnMgErLi, the corresponding numbers were 8947 and 5296. The final data reduction yielded *R*_int_ = 6.3% and *R*_int_ = 2.7%, respectively.

**Figure 5:**
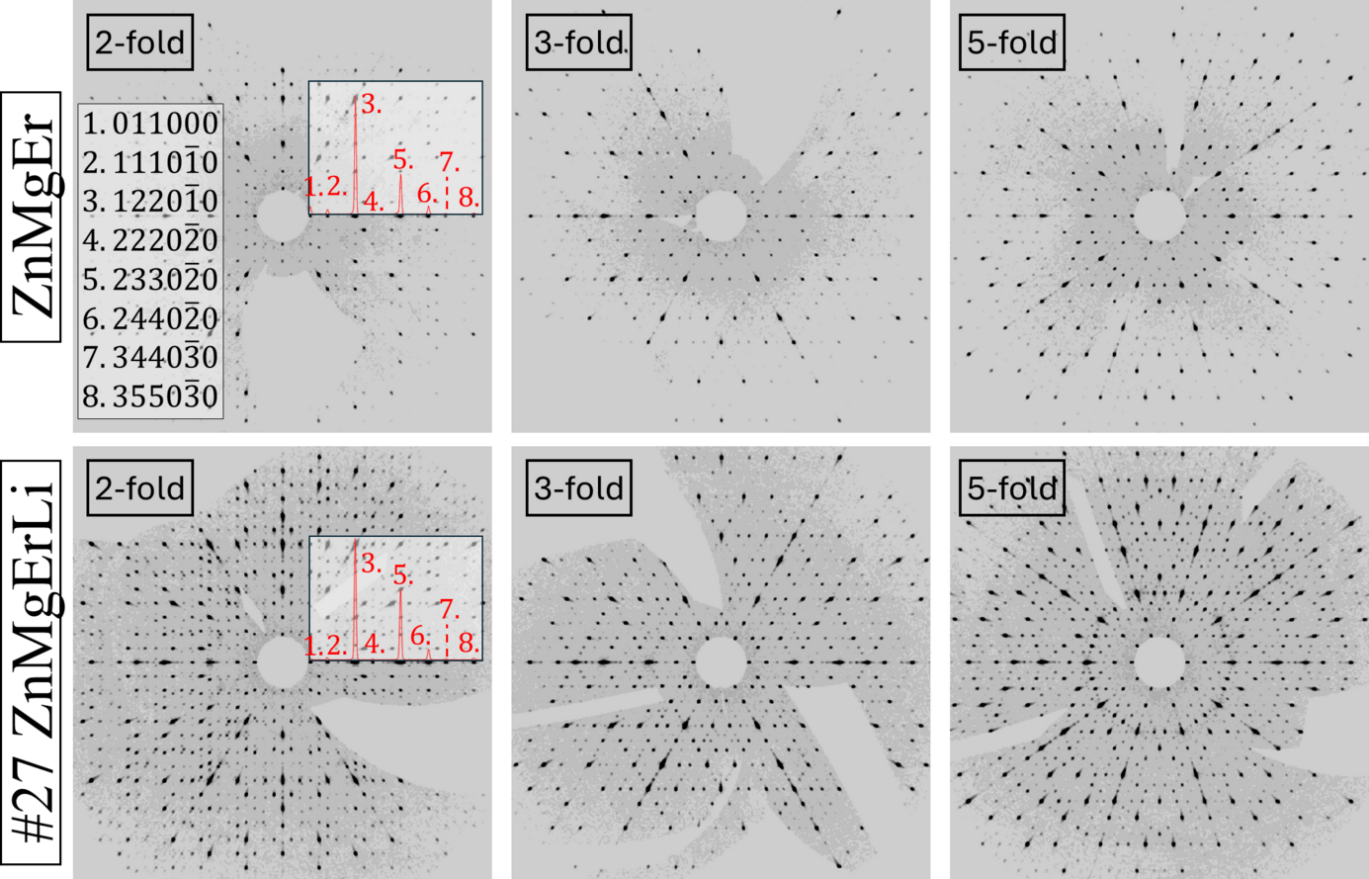
The unwarped diffraction data images in high-symmetry planes.

Three characteristic icosahedral symmetry axes were identified, and the diffraction patterns were unwarped (Fig. 5 ▸). The Li-containing sample exhibited more pronounced low-intensity reflections, though no superlattice reflections were detected. Diffuse scattering was minimal in both cases, indicating high crystal quality and the absence of correlated disorder. The PI lattice was additionally confirmed by electron diffraction (Fig. S3). In contrast to monocrystal X-ray diffraction, the electron diffraction image exhibits a linear phason strain along the twofold direction. It is therefore evident that the amount of phason strain varies in the synthesized sample.

For the twofold section, peak intensity profiles were analyzed along a selected direction for both quasicrystals. The clearest relative differences were found in the intensity ratios of peak 5 (233020) and peak 1 (011000) relative to peak 3 (122010). In ZnMgErLi, the peak 5/3 ratio was higher, and the 1/3 ratio lower compared with ZnMgEr. This confirms the PXRD observations: the enhanced 332002 reflection in Li-containing samples and the reduced 110000 reflection arise from modifications in the atomic structure due to Li substitution rather than impurity contributions. Both reflections are symmetry-equivalent to corresponding peaks in the unwarped single-crystal diffraction patterns.

### 4.1. The perpendicular-space electron density

The *ab initio* structure solution, based on single-crystal diffraction data, was carried out using *SUPERFLIP* (Palatinus & Chapuis, 2007 ▸). The crystallographic *R* factor was 22.604% for ZnMgEr and 32.919% for ZnMgErLi. The lower *R* factor for ZnMgEr reflects the use of reflections with 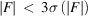, whereas for the Li-containing sample the threshold was set to 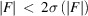. Although higher for ZnMgErLi, the values are acceptable for deriving general structural features.

To enable direct comparison of crystal features from electron-density maps, a common reflection set was extracted from both datasets. In total, 2163 symmetry-inequivalent reflections were identified and used to calculate the inverse Fourier transform. A linear regression was applied to determine the scale factor between datasets, ensuring comparable magnitudes of the transforms.

**Figure 6:**
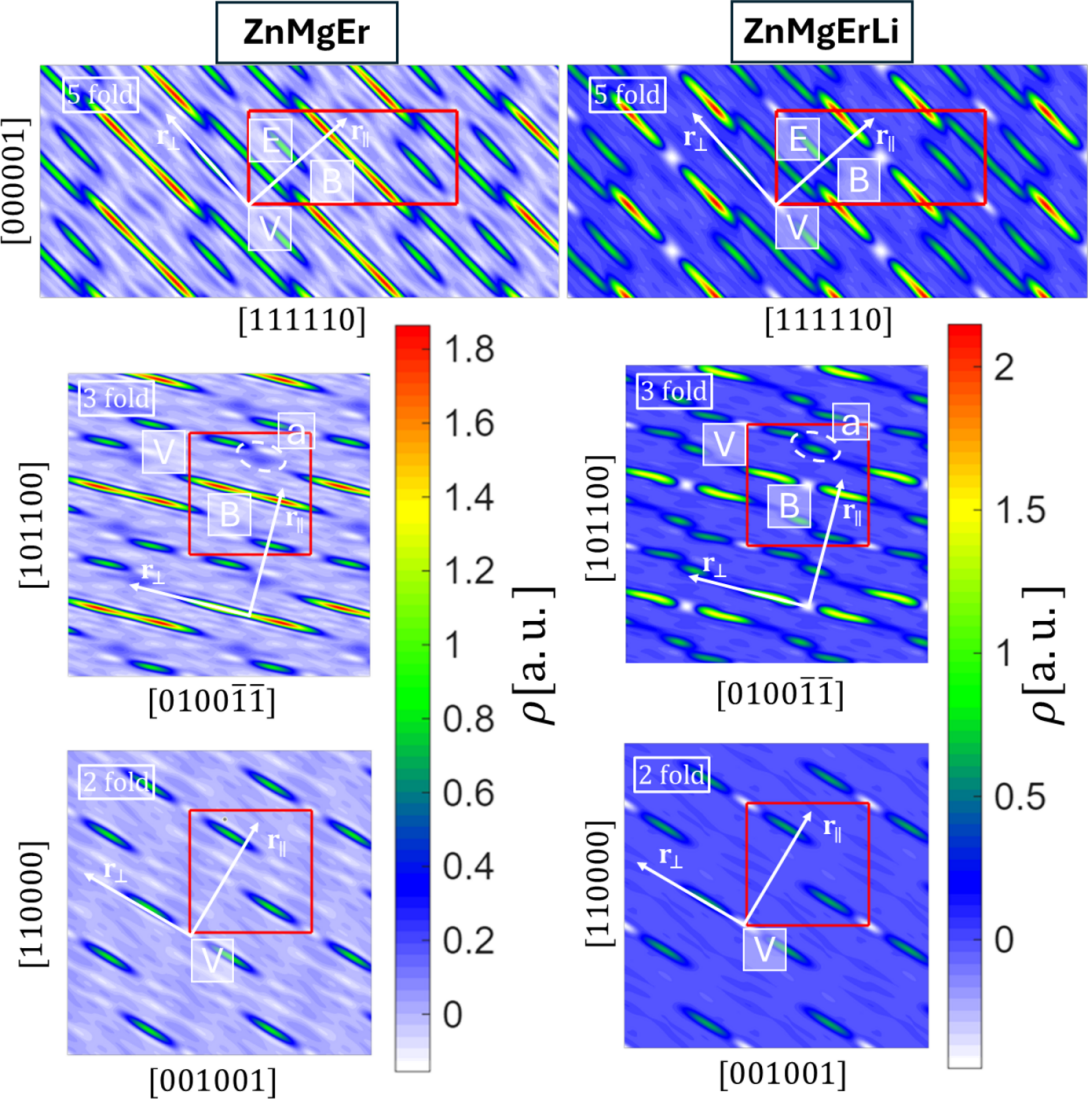
The high-symmetry 2D sections through 6D electron density in ZnMgEr and ZnMgErLi quasicrystals. Three ODs are visible with an additional empty center of the body-centered OD. The additional empty center displayed as negative electron density in ZnMgErLi is occupied by a Li atom.

Icosahedral quasicrystals can be represented as a periodic structure if the hyperspace approach is used (*e.g.* Yamamoto, 1996 ▸; Kalugin *et al.*, 1985*a* ▸; Kalugin *et al.*, 1985*b* ▸; Bak, 1985*a* ▸; Bak, 1985*b* ▸; Elser, 1986 ▸). In this higher-dimensional space, atoms are represented by compact shapes called occupation domains (ODs). Fig. 6 ▸ shows 2D sections through the 6D unit cell along planes spanned by high-symmetry axes, including the perpendicular-space component. Both quasicrystals are characterized by three ODs. The body-centered OD (OD_B_) contains the highest electron density, corresponding to the heaviest element, Er. In ZnMgEr, the center of OD_B_ is only partially occupied by Er which is manifested through the necking. If this part of the OD is associated with the real-space atomic coordinates, it corresponds to centers of rhombic triacontahedral clusters called Tsai clusters (Guo *et al.*, 2000 ▸). While ZnMgEr is classified as a Bergman-type quasicrystal, Tsai clusters can still be found in the atomic structure (Buganski & Wolny, 2023 ▸; Li *et al.*, 2008 ▸). The center of the Tsai cluster occurs in two versions where centers are either occupied by the RE element or tetrahedra (Takakura *et al.*, 2007 ▸; Euchner *et al.*, 2013 ▸; Gebresenbut *et al.*, 2020 ▸; Gebresenbut *et al.*, 2022 ▸). In ZnMgErLi, the OD_B_ center appears completely empty. However, this ‘empty’ region is most likely occupied by Li, which scatters X-rays weakly and is therefore not visible in the electron density. This suggests that Li substitutes Er at the cluster centers. The possibility that Li also replaces Mg requires further investigation.

The same feature, an apparent vacancy at the OD_B_ center, is observed in the threefold section. Additionally, a small domain (highlighted in Fig. 6 ▸ with a white ellipse, marked ‘a’) is visible adjacent to OD_V_. This domain is less pronounced in ZnMgEr but more distinct in ZnMgErLi. It originates from partially occupied sites associated with atomic phason flips along the threefold axis in real space. The low electron density indicates Mg occupancy. Similar features were reported in the Cd–Mg–Yb system (Yamada *et al.*, 2017 ▸), where the leaking domain was attributed to partially occupied sites near the dodecahedral shell of the Tsai cluster. In addition, displacement effects were observed for OD_V_ in the twofold section, where the occupancy of displaced domains varied with Mg concentration due to atom shifts along the direction perpendicular to the section plane. In our case, only the threefold section shows such concentration-dependent variation of the displaced domain.

**Figure 7:**
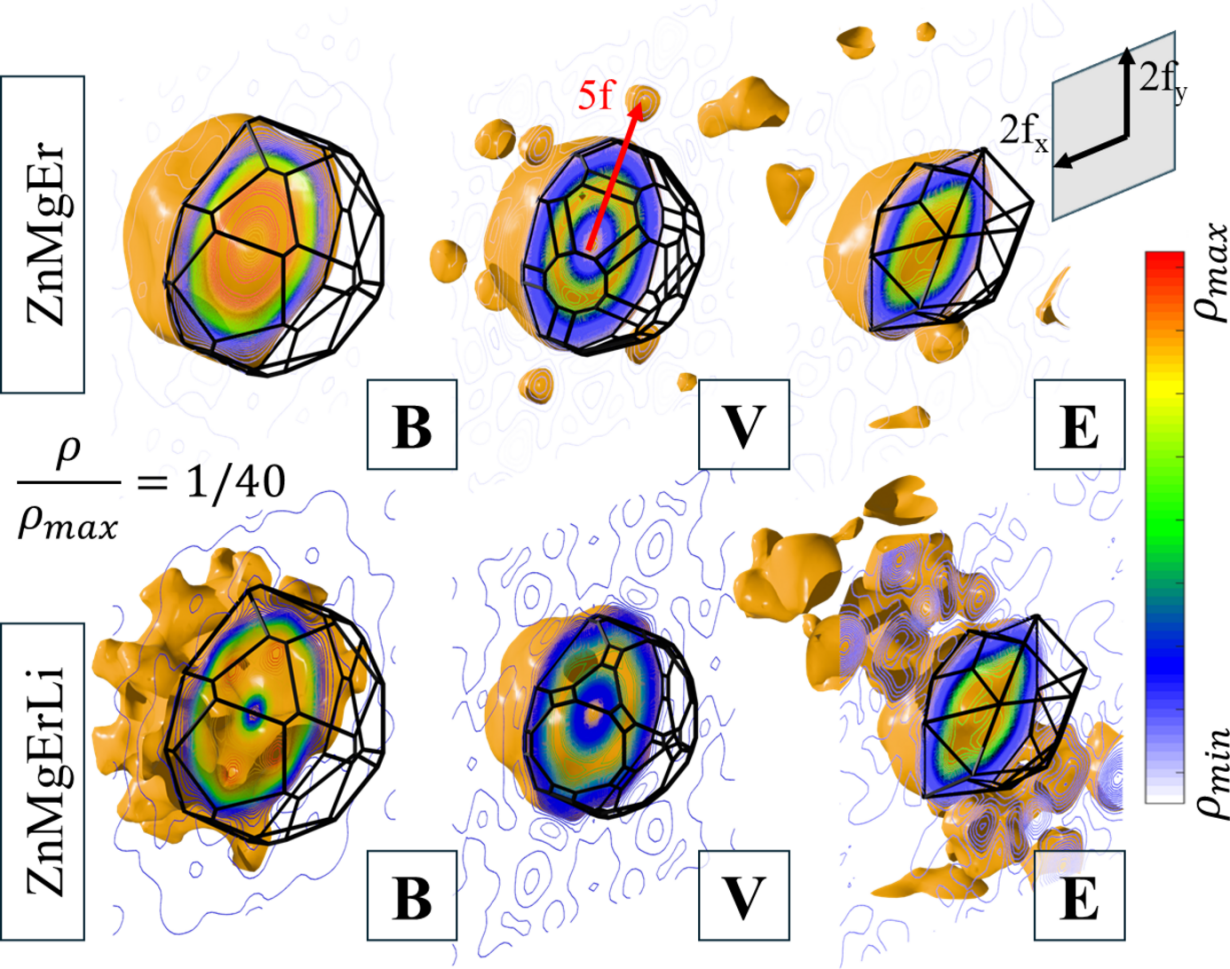
The 3D reconstruction of ODs in the perpendicular space based on the inverse Fourier transform. Isosurfaces were cut by a plane perpendicular to the twofold axis and half the shape was substituted with a wireframe approximating the OD shape.

The full 3D reconstruction of ODs can deliver even more information about the atomic structure. For that purpose, the inverse Fourier transform was calculated in the perpendicular space at the real-space coordinates corresponding to the center of each OD. Isosurface plots prepared for the value of 1/40 of the maximal electron density 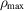 are presented in Fig. 7 ▸. Shapes of domains can be approximated by a truncated icosahedron (OD_B_), an icosahedron with vertices and edges truncated (OD_V_) and a rhombic icosahedron with vertices at the fivefold axis truncated (OD_E_). The OD_V_ has a lower level of truncation. The shapes found are different from those obtained for the so-called simple-decoration model (Henley & Elser, 1986 ▸), where atoms occupy vertices and mid-edge positions of prolate and oblate rhombohedra of the Ammann–Kramer–Neri tiling (Kramer & Neri, 1984 ▸) with additional atoms at the long body-diagonal of the prolate rhombohedron. Based on the contour plots, we find similarity to the atomic structure of AlCuLi (de Boissieu *et al.*, 1991 ▸; Guyot & Audier, 2014 ▸; Guyot *et al.*, 1990 ▸). This is expected, since it represents the same type of structure as PI ZnMgRE.

The most relevant difference between ZnMgEr and ZnMgErLi is visible for OD_V_. The ZnMgEr phase has additional domains along the fivefold symmetry axis corresponding to atomic phason flips (de Boissieu, 2008 ▸; de Boissieu, 2012 ▸). In real space, two sites are energetically favorable for occupation by the atom; therefore it can jump between those two positions, resulting in OD splitting. Due to the substitution with Li, the bond distribution changes; therefore, one of the phason-flip sites stops being occupied by an atom. The phason-flip site might be occupied by Mg which is substituted with Li, and that results in changes in the structure dynamics. The hypothesis about Mg being the flipping atom is based on the OD reconstruction for ZnMgTm, which is isostructural to ZnMgEr, where the exterior of OD_V_ is occupied by Mg (Buganski *et al.*, 2020 ▸). Additionally, the isosurface for ZnMgErLi, as shown in Fig. 7 ▸, has protruding islands along the fivefold axes which is related to small domains joining the main OD. Regarding the OD_B_, spikes at threefold axes for ZnMgErLi can come from OD_E_. Both are close to each other and cannot be fully separated in the perpendicular space. It is confirmed by large domains observed for OD_E_ of ZnMgErLi which are residuals of OD_B_. They are absent in ZnMgEr because the phase retrieval was more accurate for that phase.

### 4.2. The real-space analysis

In real space, two regions are of particular interest: the environments of the Tsai and Bergman clusters. Although ZnMgEr is a Bergman-type quasicrystal, ideal Tsai clusters with complete shell structures can still be identified. This conclusion is consistent with analyses of periodic approximant crystals (Buganski & Wolny, 2023 ▸) as well as both Bergman-type (Buganski *et al.*, 2020 ▸) and Tsai-type (Buganski *et al.*, 2024*a* ▸) quasicrystals.

**Figure 8:**
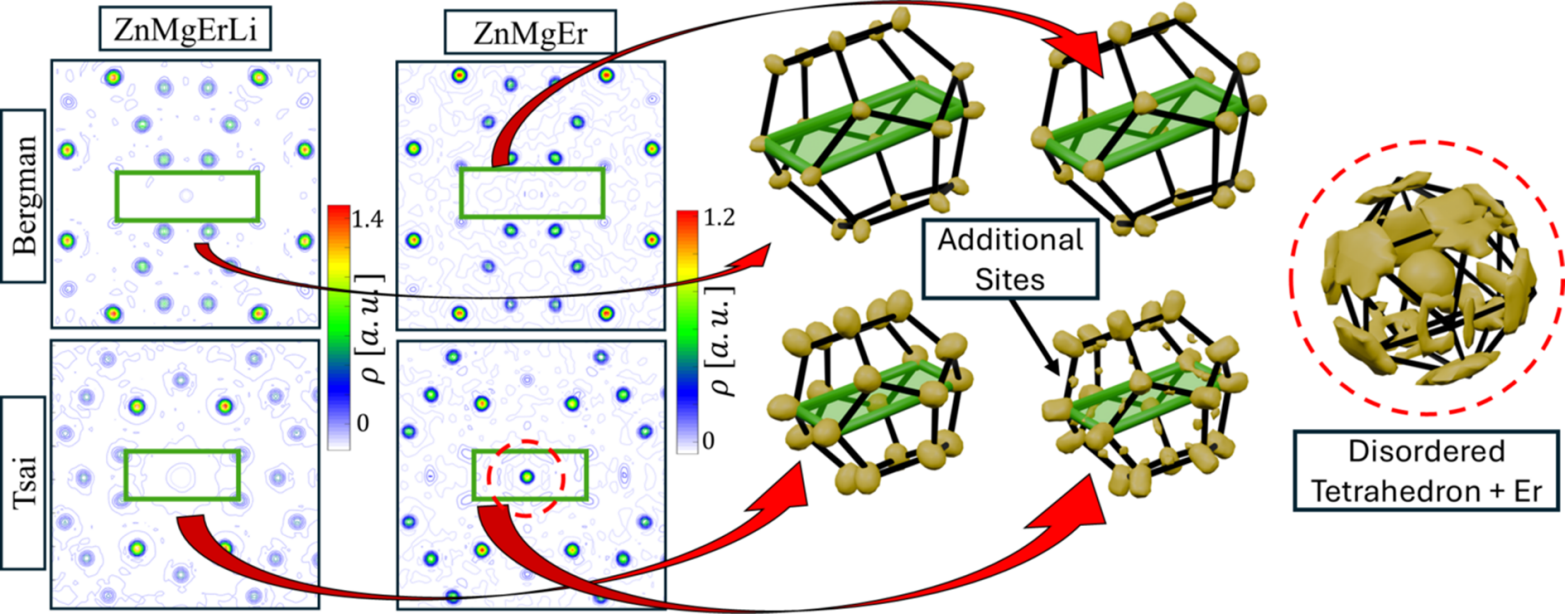
The real-space electron-density map around Bergman and Tsai clusters in ZnMgEr and ZnMgErLi quasicrystals. The substitution of Er by Li is visible as empty space in the center of the Tsai cluster. The presence of radial displacement along the threefold axis is visible for the Bergman cluster in the ZnMgErLi quasicrystal. The marker of a tetrahedron in the center of the Tsai cluster vanishes for the Li-containing phase.

Fig. 8 ▸ compares representative Tsai and Bergman clusters in ZnMgEr and ZnMgErLi. The most striking feature is the absence of electron density at the center of the Tsai cluster in ZnMgErLi, corresponding to Li substitution. In contrast, the same site in ZnMgEr is occupied by Er and surrounded by residual electron density; it exhibits icosahedral symmetry, being a manifestation of a disordered tetrahedron. The icosahedral lattice size of 5.160 (4) Å, as measured with monocrystal diffraction, allows it to host the full tetrahedron without violating steric conditions. In the Zn_7.33_Sc quasicrystal the entire tetrahedron is stored in the center of the Tsai cluster even though the lattice size is smaller (Yamada *et al.*, 2016 ▸). Markers of the remaining electron density in the center of the Tsai cluster provide evidence that the tetrahedral shell is not restricted to Tsai-type phases but also appears in Bergman-type quasicrystals. Moreover, additional electron density is observed at the edges of the nearest dodecahedral shell. This feature likely reflects atomic phason flips, where motion within the tetrahedron induces displacements of atoms on the dodecahedral shell. In ZnMgErLi, Li substitution suppresses this motion, and no tetrahedral features are detectable.

Another notable difference appears in the Bergman cluster: the isosurface at the vertices of the dodecahedral shell is slightly elongated along the threefold axis in ZnMgErLi. This feature corresponds to the displacement observed in the 2D perpendicular-space section and may result from Mg → Li substitution. The elongation is a sign of changed bonding distance between atoms.

No additional structural changes could be identified from the *ab initio* solution. However, for completeness, the supporting information (Fig. S4) includes a 100 × 100 Å real-space slab perpendicular to the twofold axis for both quasicrystals, as well as isosurfaces for all cluster shells.

## 5. Conclusions

We successfully synthesized single grains of primitive icosahedral quasicrystals in the new ZnMg(Dy, Ho, Er, Tm)Li systems. The quasicrystalline phase exhibits a unique icosahedral morphology, favoring triangular faces over pentagons. According to the Wulff principle, crystal growth along the threefold direction is the slowest. Detailed studies were carried out for Zn_62.8_Mg_28.6_RE_3.6_Li_5_ and Zn_66_Mg_24.8_RE_3.9_Li_5.3_ compositions. For Dy-containing samples with Zn-rich composition, modifications to the thermal treatment protocol may be required to obtain a stable quasicrystalline phase, and further tests are needed.

The range of Li substitution has not been fully explored, but individual samples with 3 at.% and even 10 at.% Li replacing Mg in the starting compositions were successfully grown. This wide substitution range offers a promising route towards long-range magnetically ordered Bergman-type quasicrystals. Importantly, Li substitution modifies not only the *e*/*a* relevant for magnetic ordering but also the spatial distribution of RE magnetic moments due to replacement of RE atoms in specific sites at centers of Tsai clusters. As a result, the optimal *e*/*a* ratio for inducing magnetic order may differ from that established for Tsai-type quasicrystals.

## Supplementary Material

Supporting figures. DOI: 10.1107/S205327332501099X/gau5001sup1.pdf

## Figures and Tables

**Table 1:** Details of the analyzed samples

Sample No.	Composition	Lattice constant 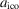 (Å)	Annealing time (h)
22	Zn_62.8_Mg_33.6_Er_3.6_	5.160 (4)†	30
			
27	Zn_62.8_Mg_28.6_Er_3.6_Li_5_	5.143 (3)†	32
35	Zn_62.8_Mg_23.6_Er_3.6_Li_10_	5.128 (4)	52
50	Zn_66_Mg_24.8_Er_3.9_Li_5.3_	5.129 (3)	75
67	Zn_66_Mg_24.8_Er_3.9_Li_5.3_	5.130 (3)	3.5
68	Zn_62.8_Mg_28.6_Er_3.6_Li_5_	5.138 (3)	3.5
			
55	Zn_62.8_Mg_28.6_Ho_3.6_Li_5_	5.141 (2)	101
56	Zn_62.8_Mg_30.6_Ho_3.6_Li_3_	5.143 (3)	101
84	Zn_66_Mg_24.8_Ho_3.9_Li_5.3_	5.142 (4)	4
			
69	Zn_66_Mg_24.8_Tm_3.9_Li_5.3_	5.123 (2)	30
70	Zn_62.8_Mg_28.6_Tm_3.6_Li_5_	5.128 (2)	30
			
72	Zn_62.8_Mg_28.6_Dy_3.6_Li_5_	5.151 (4)	30

† Lattice constant based on monocrystal X-ray diffraction.

## Data Availability

Monocrystal X-ray diffraction data can be downloaded from the Open Science Framework: sample #27 ZnMgErLi: DOI 10.17605/OSF.IO/4E9ZJ; sample #51 ZnMgEr: DOI 10.17605/OSF.IO/297KW. Remaining data can be obtained upon request to ireneusz.buganski@fis.agh.edu.pl.
