# Supplementary material for: Primitive icosahedral quasicrystals in ZnMgLi(Dy, Ho, Er, Tm) systems
Source: Acta Crystallogr A Found Adv. 2026 Jan 22;82(Pt 2):108–17. doi: 10.1107/S205327332501099X (PMC12954479; doi:10.1107/S205327332501099X)
Supplement: Supplementary file 1 [file a-82-00108-sup1.pdf]

FOUNDATIONS
ADVANCES

Volume 82 (2026)

Supporting information for article:

Primitive icosahedral quasicrystals in ZnMgLi(Dy, Ho, Er, Tm) sysytems

Ireneusz Buganski, Stanislav Vrtnik, Radoslaw Strzalka, Andreja Jelen, Sandra Drev, Janusz Wolny and Nobuhisa Fujita

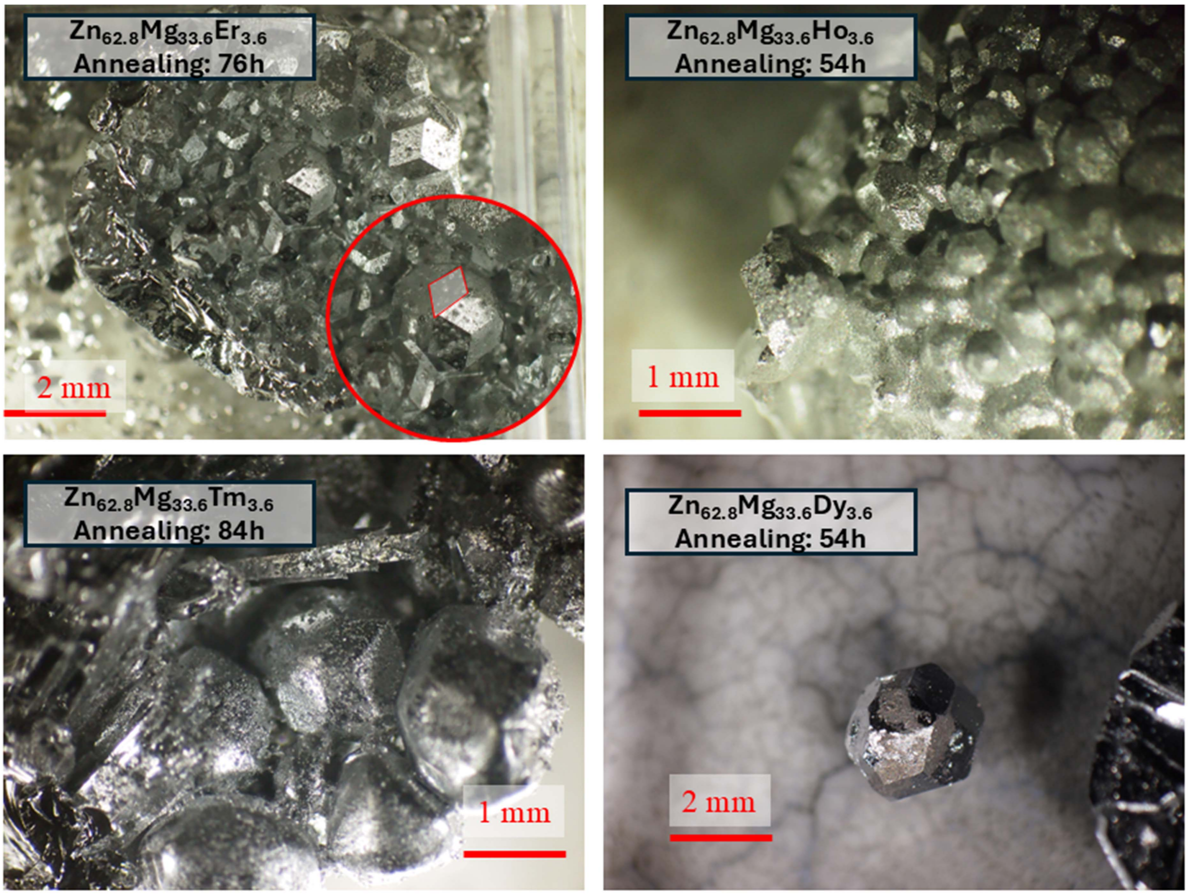

Figure S1 Samples of ZnMgRE (RE=Dy, Ho, Er, Tm) with primitive icosahedral lattice. Grains with rhombic triacontahedral morphology are visible.

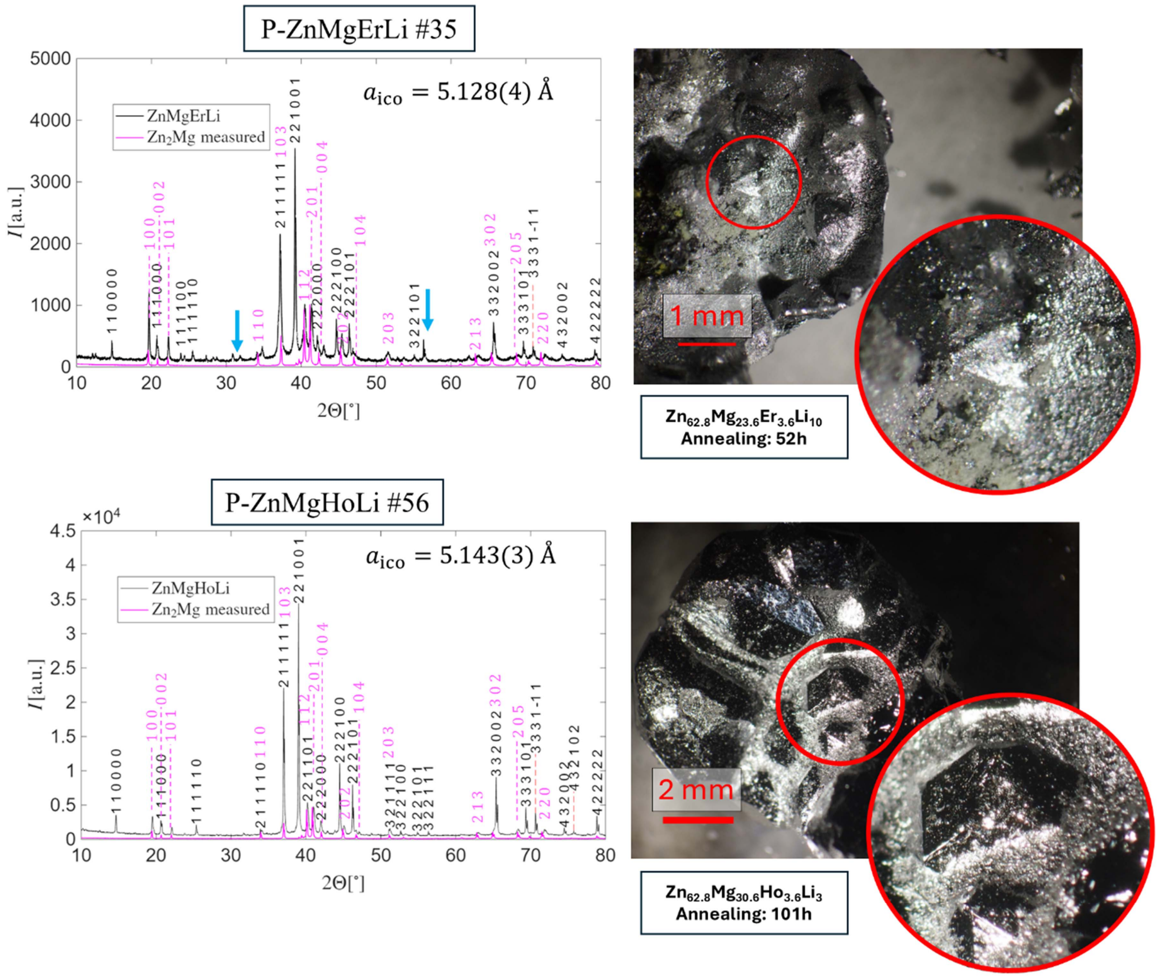

Figure S2 The powder X-ray diffraction pattern of Sample #35 in ZnMgErLi and sample #56 in ZnMgHoLi system. The sample constains respectively 10 at. % and 3 at. % of Li. After heat treatment, triangular shapes can be seen at grains of Er-containing sample but rhombuses for the sample with Ho. Higher content of Li causes change in the morphology.

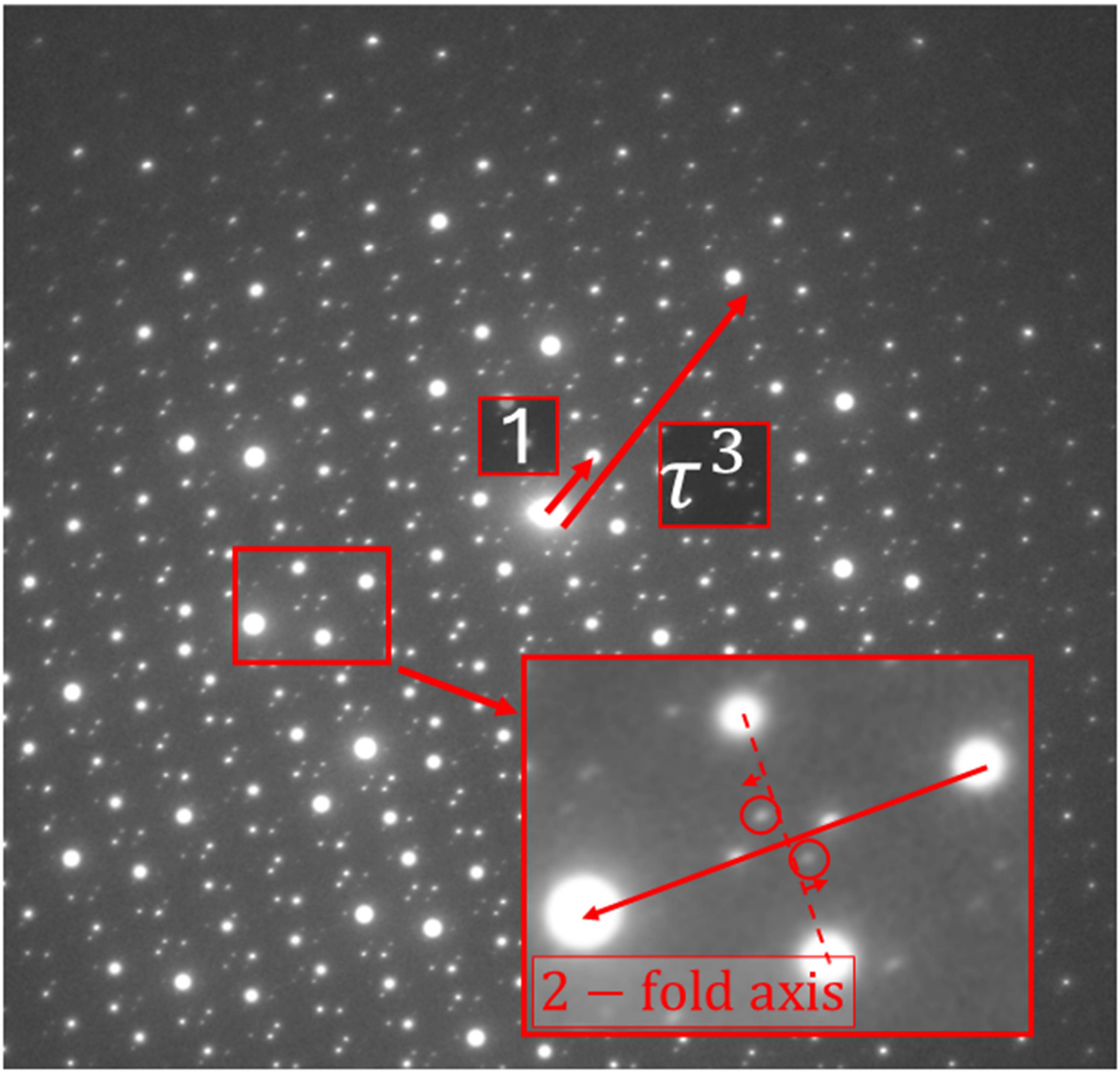

Figure S3 Electron diffraction pattern in plane perpendicular to 2-fold symmetry axis. The scaling characteristic to primitive icosahedral quasicrystal is indicated by the presence of $\tau^3, \tau = (1 + \sqrt{5})/2$ peaks. No superstructure reflections are found. The linear phason strain occurs along 2-fold axis as evident by shifted position of peaks.

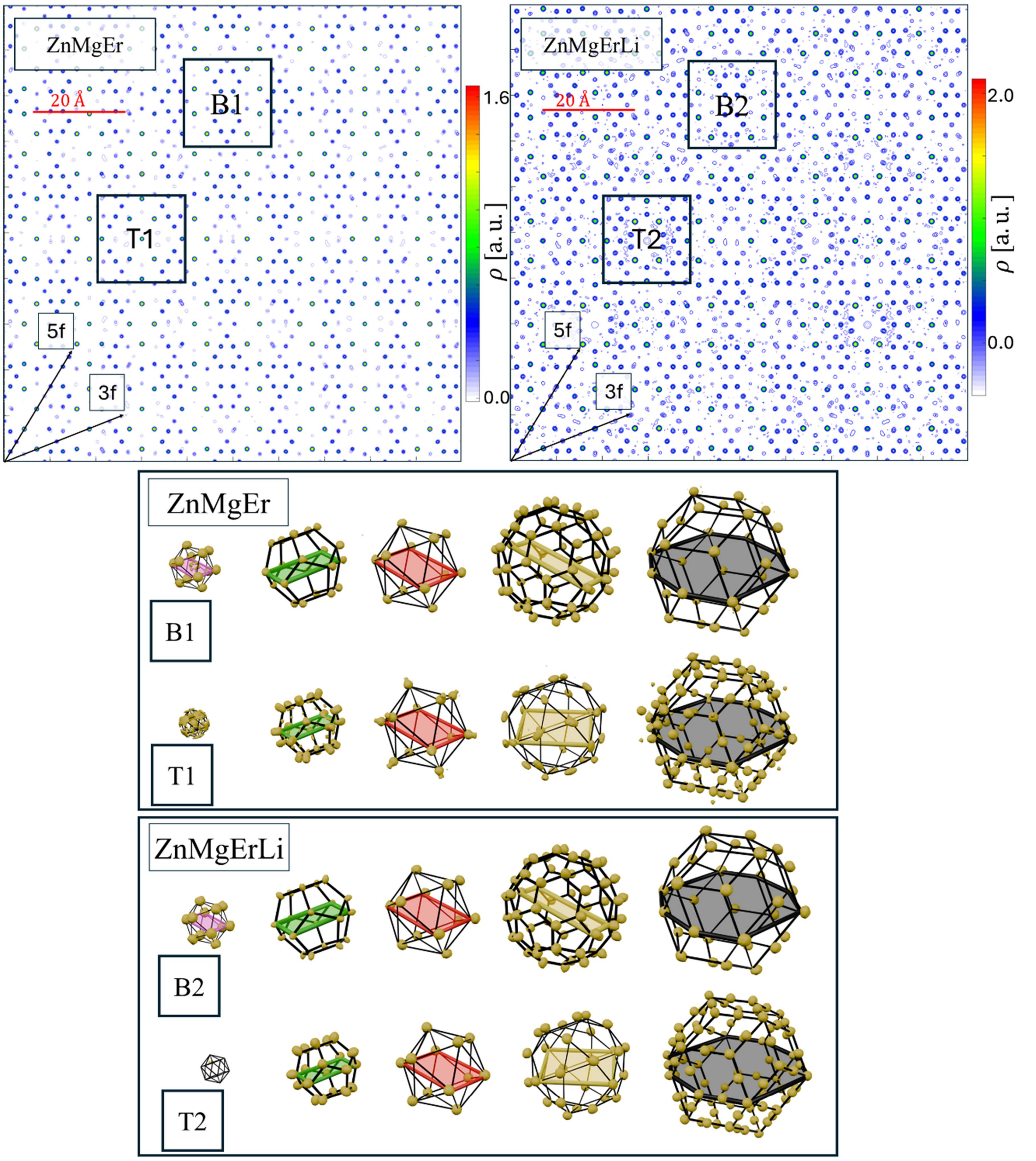

Figure S4 The 2D section through 3D electron density map in a real space. Isosurface plots for selected clusters are shown.
